# Blockade of c-Met-Mediated Signaling Pathways by E7050 Suppresses Growth and Promotes Apoptosis in Multidrug-Resistant Human Uterine Sarcoma Cells

**DOI:** 10.3390/ijms232314884

**Published:** 2022-11-28

**Authors:** Tsung-Teng Huang, Chuan-Mu Chen, Ying-Wei Lan, Song-Shu Lin, Kong-Bung Choo, Kowit-Yu Chong

**Affiliations:** 1 Department of Medical Biotechnology and Laboratory Science, College of Medicine, Chang Gung University, Taoyuan 33302, Taiwan; 2 Graduate Institute of Biomedical Sciences, Division of Biotechnology, College of Medicine, Chang Gung University, Taoyuan 33302, Taiwan; 3 Department of Life Sciences, Agricultural Biotechnology Center, National Chung Hsing University, Taichung 402, Taiwan; 4 The iEGG and Animal Biotechnology Center and the Rong Hsing Research Center for Translational Medicine, National Chung Hsing University, Taichung 402, Taiwan; 5 Division of Pulmonary Biology, The Perinatal Institute of Cincinnati Children’s Research Foundation, Cincinnati, OH 45229, USA; 6 Department of Nursing, Chang Gung University of Science and Technology, Taoyuan 33302, Taiwan; 7 Hyperbaric Oxygen Medical Research Lab, Bone and Joint Research Center, Linkou Chang Gung Memorial Hospital, Taoyuan 33305, Taiwan; 8 Centre for Stem Cell Research, Faculty of Medicine and Health Sciences, Universiti Tunku Abdul Rahman, Kajang 43000, Selangor, Malaysia; 9 Department of Traditional Chinese Medicine, Chang Gung Memorial Hospital at Keelung, Keelung City 20401, Taiwan

**Keywords:** E7050, multidrug-resistant uterine sarcoma, apoptosis, cell cycle arrest, c-Met

## Abstract

E7050 is a potent inhibitor of c-Met receptor tyrosine kinase and has potential for cancer therapy. However, the underlying molecular mechanism involved in the anti-cancer property of E7050 has not been fully elucidated. The main objective of this study was to investigate the anti-tumor activity of E7050 in multidrug-resistant human uterine sarcoma MES-SA/Dx5 cells in vitro and in vivo, and to define its mechanisms. Our results revealed that E7050 reduced cell viability of MES-SA/Dx5 cells, which was associated with the induction of apoptosis and S phase cell cycle arrest. Additionally, E7050 treatment significantly upregulated the expression of Bax, cleaved PARP, cleaved caspase-3, p21, p53 and cyclin D1, while it downregulated the expression of survivin and cyclin A. On the other hand, the mechanistic study demonstrated that E7050 inhibited the phosphorylation of c-Met, Src, Akt and p38 in HGF-stimulated MES-SA/Dx5 cells. Further in vivo experiments showed that treatment of athymic nude mice carrying MES-SA/Dx5 xenograft tumors with E7050 remarkably suppressed tumor growth. E7050 treatment also decreased the expression of Ki-67 and p-Met, and increased the expression of cleaved caspase-3 in MES-SA/Dx5 tumor sections. Therefore, E7050 is a promising drug that can be developed for the treatment of multidrug-resistant uterine sarcoma.

## 1. Introduction

Uterine sarcoma is a rare type of lethal gynecologic malignancy, accounting for 3–7% of all uterine cancers. According to the World Health Organization (WHO) classification, uterine sarcoma tumor types include the most common uterine leiomyosarcoma, endometrial stromal sarcoma and undifferentiated uterine sarcoma [1,2,3]. In general, surgical resection, radiation therapy, chemotherapy, targeted therapy and hormone therapy alone or in combination are the main treatment approaches. However, these therapies for uterine sarcoma have been reported as ineffective in prolonging survival and preventing recurrence in patients [4,5]. Moreover, concerning chemotherapy, the resistance and the severe toxicity of chemotherapeutic drugs remain major impediments for successful chemotherapy. Multidrug resistance (MDR) is characterized by simultaneous development of resistance to various structurally and functionally cancer therapeutic drugs [6]. MDR is mediated by the overexpression of P-glycoprotein (P-gp), which increases transport of the various anti-cancer drugs out of cells, resulting in decreased cellular accumulation of the drugs and subsequently reduced therapeutic efficacy [7,8,9]. Therefore, there exists a need to explore high-efficiency and low-side-effect drugs to treat multidrug-resistant uterine sarcoma.

Hepatocyte growth factor/scatter factor (HGF/SF) is a multifunctional cytokine and a natural ligand of c-Met. HGF binds to and activates the transmembrane tyrosine kinase receptor, c-Met, triggering the signaling cascades involved in diverse biological activities such as cell growth, proliferation, migration, motility, survival, wound healing and angiogenesis [10,11,12]. HGF consists of a heterodimeric structure including two chains, α and β chains, by which it can bind to the receptor c-Met and induce c-Met dimerization and phosphorylation of specific tyrosine residues. This creates docking sites for adaptor proteins, such as growth factor receptor-bound protein 2 (Grb2), Grb2-associated binding protein 1 (Gab1) and src homology domain c-terminal adaptor homolog (Shc), and subsequently recruits and activates various downstream protein kinases, including phosphoinositide 3-kinase/protein kinase B (PI3K/Akt), mitogen-activated protein kinases (MAPKs), phospholipase C gamma (PLCγ) and focal adhesion kinase (FAK), among others [13,14,15,16]. It has been proved that aberrant activation of HGF/c-Met signaling can result in the induction of cell proliferation, invasion, metastasis, tumor angiogenesis and drug resistance of tumor cells in many types of cancers [17,18]. In addition, our previous study has demonstrated that transfection of c-Met shRNA into multidrug-resistant human uterine sarcoma MES-SA/Dx5 cells shuts down HGF/c-Met signaling, leading to the reduction of cell viability and the induction of apoptotic cell death [19]. Accordingly, inhibition of the HGF/c-Met signaling pathway has been considered an effective approach for cancer therapy.

Golvatinib (E7050), an orally bioavailable small molecule, is a tyrosine kinase inhibitor (TKI) with dual action against both c-Met and VEGFR2 [20]. E7050 showed potent anti-tumor activity against gastric cancer, and prolonged the lifespan of tumor-bearing mice without adverse effects [20]. Previous studies have shown that E7050 may overcome HGF-induced resistance in lung cancer cells carrying epidermal growth factor receptor (EGFR) mutations by blocking the Met/Gab1/PI3K/Akt signaling pathway. E7050 in combination with gefitinib also resulted in marked regression of tumor growth [21]. In addition, the combination of the mutant-selective epidermal growth factor receptor tyrosine kinase inhibitor (EGFR-TKI) WZ4002 and the mutant-selective Met-TKI E7050 was able to inhibit the growth of erlotinib-resistant lung tumor caused by the gatekeeper T790M mutation, Met amplification and HGF overexpression [22]. However, the underlying mechanism by which E7050 exerts its effects on multidrug-resistant uterine sarcoma has not been fully elucidated.

This research was to evaluate the cytotoxic effects of E7050 on multidrug-resistant human uterine sarcoma and ovarian cancer cells, and doxorubicin (DOX)-resistant human breast cancer cells, and to identify the molecular mechanism through which E7050 exerts its effects. We demonstrated that E7050 exhibits profound anti-tumor activity and this effect is accompanied by inhibiting the activation of c-Met and its downstream signaling effectors. The results of the present study shed light on the molecular mechanisms underlying the anti-cancer effect of E7050 in multidrug-resistant uterine sarcoma and may provide new insights into efforts for the treatment of multidrug-resistant uterine sarcoma and other cancers in the future.

## 2. Results

### 2.1. E7050 Inhibits Viability and Induces Apoptosis and S Phase Cell Cycle Arrest in MES-SA/Dx5 Cells

**Figure 1:**
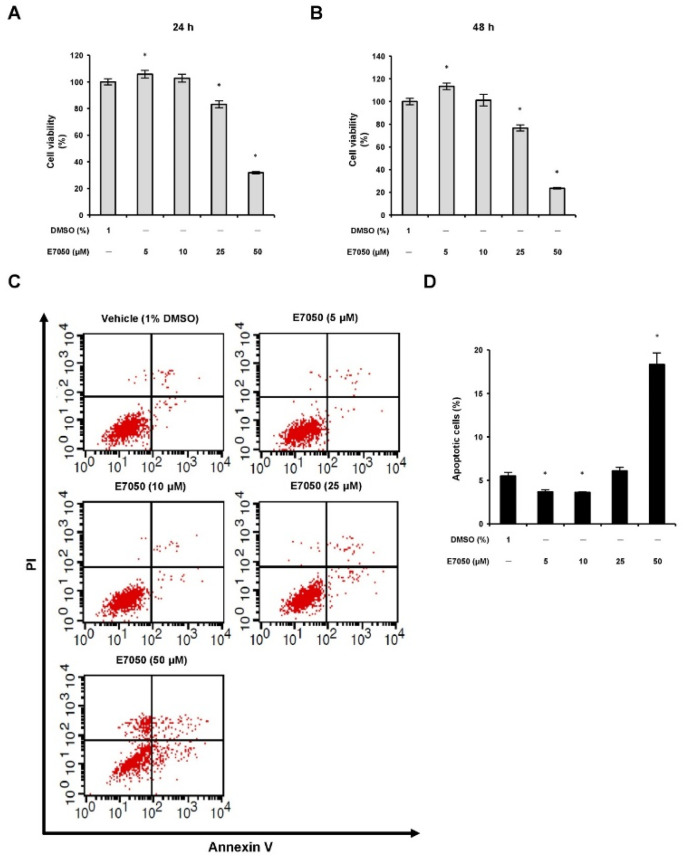
Effects of E7050 on cell viability and cell death in multidrug-resistant human uterine sarcoma MES-SA/Dx5 cells. Cells were exposed to different concentrations (5, 10, 25 and 50 μM) of E7050 for (**A**) 24 h or (**B**) 48 h, and cell viability was analyzed using the MTT assay. (**C**) Cells were treated with the indicated concentrations of E7050 for 24 h and stained with Annexin V-FITC and PI prior to flow cytometric analysis. Apoptotic cells were defined as Annexin V+/PI− plus Annexin V+/PI+ cells. (**D**) The percentages of apoptotic cells in E7050-treated cells are shown in the histogram. Data are presented as the mean ± SEM of three independent experiments. * *p* < 0.05 versus vehicle-treated control cells.

To first evaluate the effect of E7050 on the viability of multidrug-resistant human uterine sarcoma MES-SA/Dx5 cells, cells were treated with E7050 at increasing concentrations ranging from 5 to 50 μM for 24 or 48 h, and MTT assay was executed to determine cell proliferation rate. E7050 treatment caused a significant dose-dependent and time-dependent inhibition effect on the viability of MES-SA/Dx5 cells (Figure 1A,B). To investigate whether E7050 exerted its inhibitory consequence against MES-SA/Dx5 cells through cell apoptosis, an Annexin V-FITC/PI binding assay was conducted. The results showed that E7050 treatment at 50 μM remarkably increased apoptosis rate to 18.3% (Figure 1C,D). In addition, we examined the effect of E7050 on the cell cycle phase distribution in MES-SA/Dx5 cells by using a flow cytometer. As illustrated in Supplementary Figure S1A, E7050 treatment obviously increased the proportion of cells in the S phase and decreased the cell proportion in the G0/G1 phase as compared to the vehicle-treated group; suggesting E7050 could also disturb the cell cycle distribution of MES-SA/Dx5 cells. Moreover, forty-eight hours of E7050 treatment at 25 μM significantly increased the proportion of cells in sub-G1 phase when compared with the vehicle control (Supplementary Figure S1B). Taken together, these results strongly indicate that E7050 reduces the viability of MES-SA/Dx5 cells by inducing apoptosis and S phase cell cycle arrest.

### 2.2. E7050 Regulates Expression of Apoptosis-Related Proteins in MES-SA/Dx5 Cells

**Figure 2:**
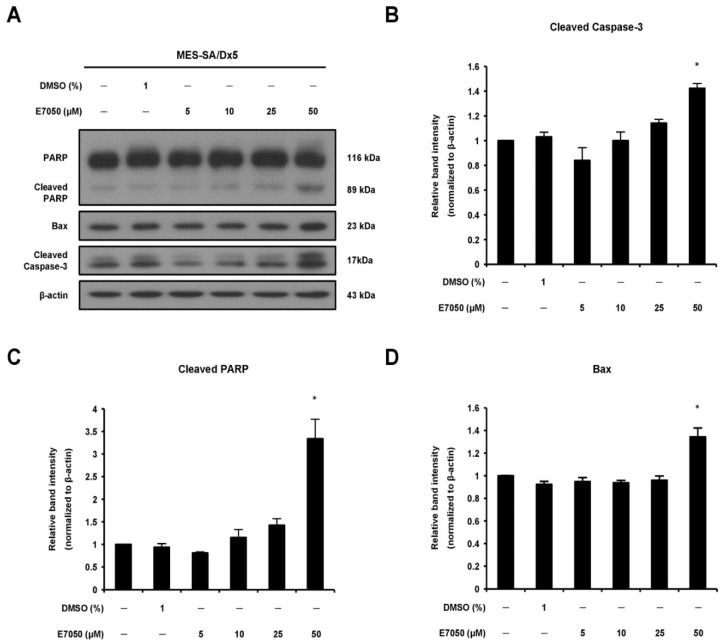
Effects of E7050 on the expression of apoptosis-related proteins in MES-SA/Dx5 cells. (**A**) Cells were treated with E7050 at various concentrations (5–50 μM) for 24 h. Cellular extracts were prepared and subjected to Western blotting with specific antibodies which recognized PARP, cleaved PARP, cleaved caspase-3, Bax and β-actin. β-actin was used as an internal loading control. The relative protein levels of (**B**) cleaved caspase-3, (**C**) cleaved PARP and (**D**) Bax were quantified by densitometry and normalized to β-actin. Data are presented as the mean ± SEM of three independent experiments. * *p* < 0.05 versus vehicle-treated control cells.

Activation of caspase-3 plays a pivotal role in the execution of apoptosis [23]. To further comprehend the apoptotic process in MES-SA/Dx5 cells, the expression patterns of caspase-3 and its downstream substrate poly (ADP-ribose) polymerase (PARP) were examined using Western blot analysis. E7050 treatment triggered the appearance of the active form caspase-3 in a dose-dependent manner (Figure 2A,B). The effector molecule PARP was also cleaved into 89 kDa fragments after E7050 treatment in MES-SA/Dx5 cells (Figure 2A,C). Additionally, to investigate the possible role of Bcl-2 family members in apoptosis induced by E7050, Western blot analysis was performed to assess the effect of E7050 on the expression of pro-apoptotic protein Bax. E7050 treatment at a concentration of 50 μM markedly enhanced the expression level of Bax in MES-SA/Dx5 cells (Figure 2A,D). Hence, we speculate that E7050-induced apoptosis of multidrug-resistant cancer cells is mediated through the caspase-3-dependent pathway.

### 2.3. E7050 Modulates the Expression Levels of Survivin, P21 and P53 in MES-SA/Dx5 Cells

**Figure 3:**
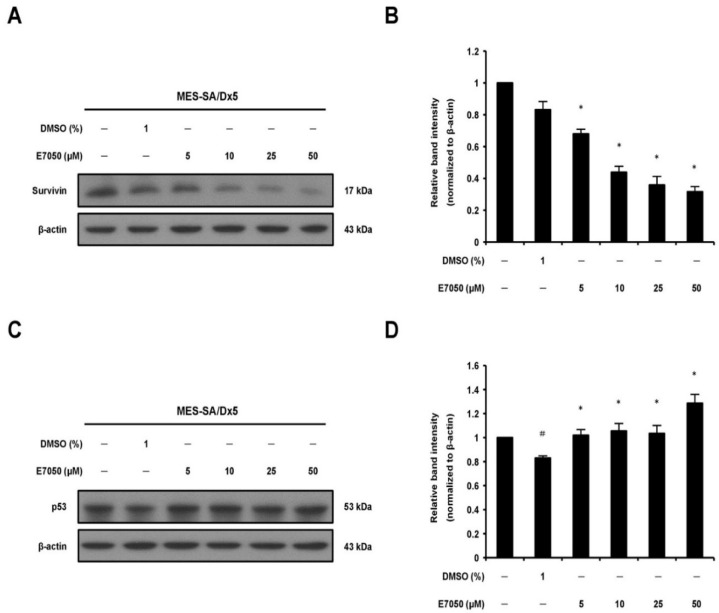
Effects of E7050 on the expression of survivin and p53 proteins in MES-SA/Dx5 cells. (**A**) Cells were treated with E7050 at various concentrations (5–50 μM) for 6 h; thereafter, survivin protein expression level was measured by Western blot analysis. β-actin was used as an internal loading control. (**B**) The relative protein level of survivin was quantified by densitometry and normalized to β-actin. (**C**) Cells were treated for 24 h with different concentrations of E7050, and Western blotting was performed to assess p53 protein expression. (**D**) The relative protein level of p53 was quantified by densitometry and normalized to β-actin. Data are presented as the mean ± SEM of three independent experiments. ^#^ *p* < 0.05 versus untreated cells. * *p* < 0.05 versus vehicle-treated control cells.

Survivin, a predominant member of the inhibitor of apoptosis protein (IAP) family, has been detected in a variety of human cancers and is related to the progression of malignant tumors; meanwhile, survivin can inhibit caspase-3 activation and thereby prevent apoptosis [24,25]. Accordingly, Western blot analysis was performed to detect the expression level of survivin in MES-SA/Dx5 cells, and found that E7050 treatment effectively decreased the protein level of survivin in a dose-dependent manner as shown in Figure 3A,B. Next, p53 and p21 have been found to be importantly involved in apoptosis and cell cycle induced by a broad range of agents [26,27]. To access the effect of E7050 on the protein expression levels of p53 and p21 in MES-SA/Dx5 cells, we performed Western blot analysis. The data showed that E7050 treatment for 24 h induced the upregulation of p53 protein expression compared with the vehicle control (Figure 3C,D). Furthermore, a similar increase in p21 protein expression was observed in MES-SA/Dx5 cells after E7050 treatment for 48 h (Supplementary Figure S1F,H). Overall, these results implicate that E7050 may exert anti-tumor effects by downregulating the expression of survivin, and upregulating the expression of p53 and p21.

### 2.4. E7050 Has No Inhibitory Effect on HGF Production in MES-SA/Dx5 Cells

**Figure 4:**
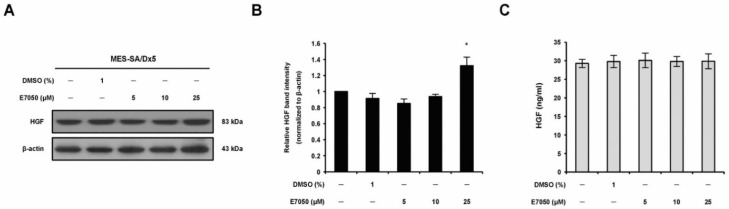
Effects of E7050 on the expression and secretion of HGF in MES-SA/Dx5 cells. (**A**) Cells were treated with various concentrations (5–25 μM) of E7050 for 24 h. Whole cell extracts were prepared and subjected to Western blotting using antibodies against HGF and β-actin. β-actin was used as an internal loading control. (**B**) Densitometric analysis of blots relative to HGF protein after normalization with β-actin. (**C**) Secreted HGF in cell culture media was determined by ELISA. Data are presented as the mean ± SEM of three independent experiments. * *p* < 0.05 versus vehicle-treated control cells.

To evaluate the effect of E7050 on the protein expression of HGF in MES-SA/Dx5 cells, we performed Western blot analysis. In 5 and 10 μM E7050-treated cells, HGF protein expression was similar in comparison to the vehicle control, whereas high HGF expression was observed in 25 μM E7050-treated cells (Figure 4A,B). To further assess the production/secretion of HGF by E7050-treated cells, we measured the levels of HGF protein in the cell culture supernatants by ELISA. Results showed that the levels of HGF secretion in the cell culture medium were not evidently changed in E7050-treated cells compared with the matched control, as shown in Figure 4C. These observations implicate that HGF is not the target of E7050 and c-Met inhibitor may overcome high expression level of HGF in MES-SA/Dx5 cells. According to the previous studies, E7050 is an active c-Met tyrosine kinase inhibitor and has been shown to inhibit the phosphorylation of c-Met induced by HGF.

### 2.5. E7050 Inhibits the Phosphorylation of c-Met and Downstream Signaling Effectors in HGF-Stimulated MES-SA/Dx5 Cells

**Figure 5:**
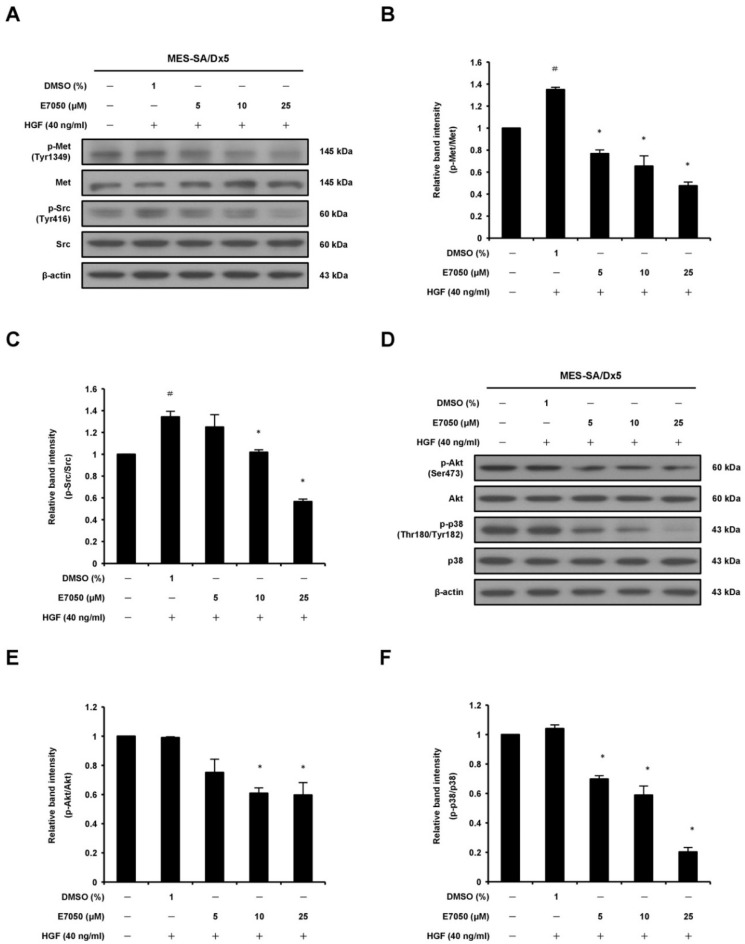
Effects of E7050 on the phosphorylation of c-Met and its downstream signaling mediators in HGF-stimulated MES-SA/Dx5 cells. Cells were starved in serum-free medium for 24 h, pretreated with various concentrations (5–25 μM) of E7050 for 1 h and then stimulated with HGF (40 ng/mL) for 10 min (c-Met and Src) or 30 min (Akt and p38 MAPK) before protein extraction. The expression levels of Met and its downstream intracellular kinases and their phosphorylated forms were evaluated using Western blot analysis. β-actin was used as an internal loading control. (**A**) E7050 inhibited the phosphorylation of c-Met and Src in HGF-induced MES-SA/Dx5 cells. The images shown are representative Western blotting data. (**B**) The ratio between the expression of Met and p-Met was calculated and is shown. (**C**) The ratio between the expression of Src and p-Src was calculated and is shown. (**D**) E7050 suppressed the phosphorylation of Akt and p38 MAPK in HGF-induced MES-SA/Dx5 cells. The images shown are representative Western blotting data. The compiled results of the ratios of p-Akt (**E**) and p-p38 MAPK (**F**) normalized to relative protein levels are shown. Data are presented as the mean ± SEM of three independent experiments. ^#^ *p* < 0.05 versus untreated cells. * *p* < 0.05 versus vehicle-treated control cells.

To confirm whether E7050 exerts potent anti-tumor effect by targeting c-Met-mediated signaling pathways, we next detected the expression of c-Met and its downstream effectors in HGF-stimulated MES-SA/Dx5 cells. Cells were treated with the indicated concentrations of E7050, followed by the addition of 40 ng/mL HGF, and the protein levels of c-Met and downstream signaling kinases along with their phosphorylated forms were examined using Western blot analysis. As shown in Figure 5A,B, we found that E7050 treatment resulted in a remarkable inhibition of HGF-stimulated phosphorylation of c-Met. Moreover, E7050 also effectively suppressed the phosphorylation of downstream signal transduction proteins such as Src, Akt and p38 MAPK in a concentration-dependent manner in MES-SA/Dx5 cells without affecting the expression of total Src, Akt and p38 MAPK proteins (Figure 5A,C–F). However, the levels of p-JNK and p-ERK proteins were not inhibited by the E7050 treatment compared with the control group. Similar results were observed in HUVECs. HGF-induced phosphorylation of c-Met, as well as the activation of its downstream signaling proteins, including p-FAK, p-Akt, p-JNK, p-ERK and p-p38 MAPK, was inhibited by E7050 in a dose-dependent manner (Supplementary Figure S2). These results indicate that E7050 has an inhibitory impact on the activation of HGF-c-Met signaling pathways in MES-SA/Dx5 cells and HUVECs, thereby providing the molecular mechanisms by which E7050 exhibits anti-cancer effect in MES-SA/Dx5 cells.

### 2.6. E7050 Suppresses Tumor Growth and c-Met Phosphorylation in the MES-SA/Dx5 Tumor Xenograft Model

**Figure 6:**
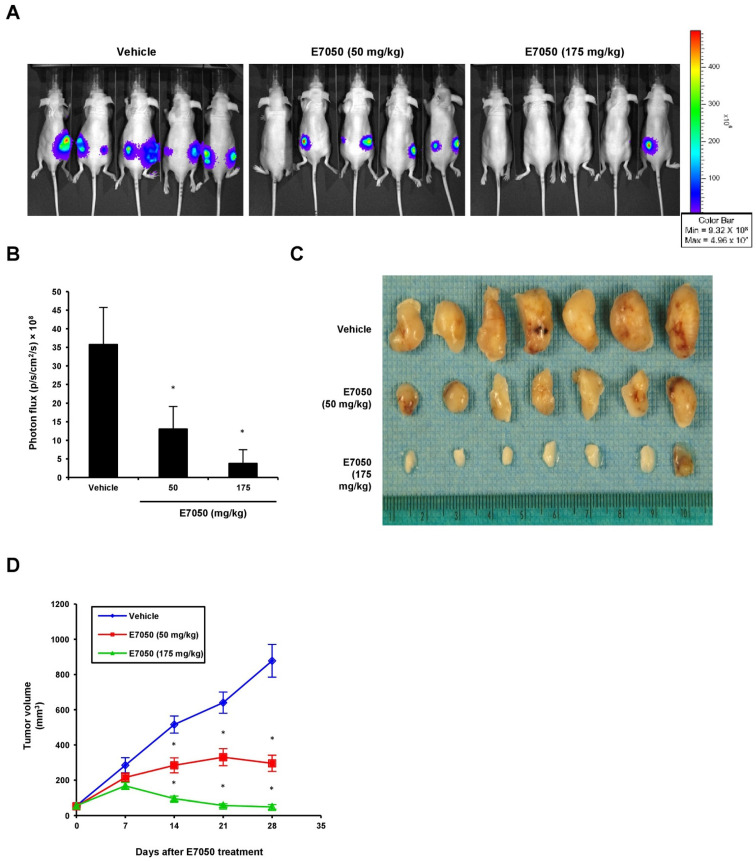
Effects of E7050 on the growth of MES-SA/Dx5 subcutaneous xenograft tumors in nude mice. Nude mice bearing MES-SA/Dx5-LG xenograft tumors were treated daily with vehicle or E7050 at 50 and 175 mg/kg for 28 days. (**A**) Representative bioluminescence images of five mice from each group are shown using an IVIS Imaging System. The color scale represents photon flux (photons/s/cm^2^/steradian). (**B**) Emitted bioluminescence (p/s/cm^2^/s) from vehicle- and E7050-treated mice representing tumor growth at day 28. (**C**) The image shows the tumor size of MES-SA/Dx5 xenografts from vehicle- and E7050-treated groups at the end of the experiment. (**D**) Volume (mm^3^) of developing MES-SA/Dx5 xenograft tumors in vehicle- and E7050-treated mice was measured at days 7, 14, 21 and 28. Data are expressed as the mean ± SEM (*n* = 5 per group). * *p* < 0.05 compared with the vehicle control group.

**Figure 7:**
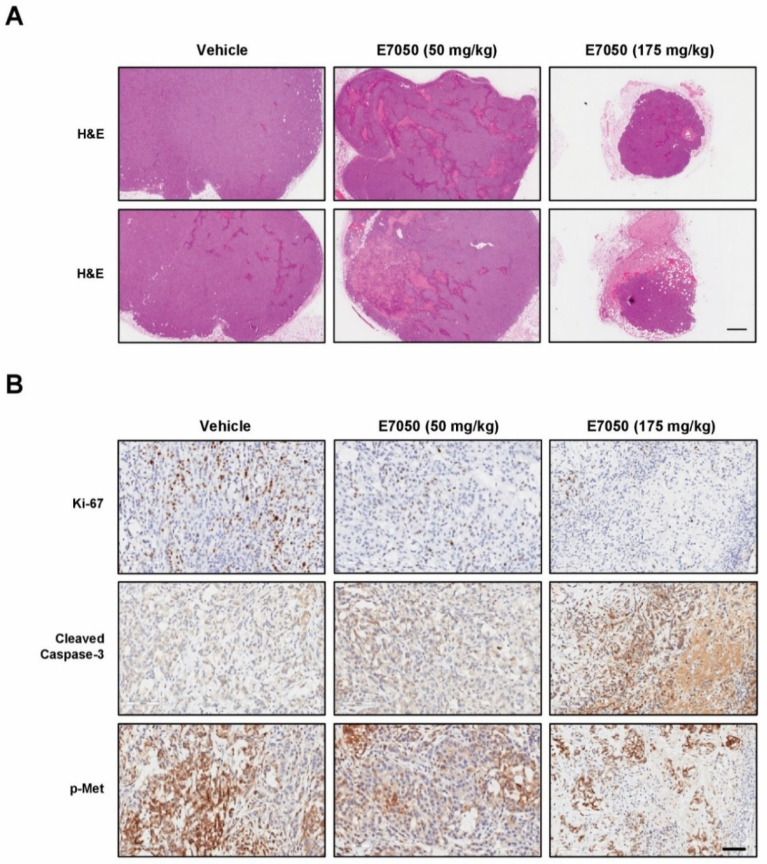
Effects of E7050 on histology and the expression levels of caspase-3 p17, Ki-67 and p-Met in tumor tissue sections obtained from MES-SA/Dx5 cell line-derived xenografts. (**A**) Representative photographs of tumor tissue sections from each group stained with H&E. Scale bar, 500 μm. (**B**) Immunohistochemical analysis was used to analyze cell proliferation marker Ki-67, apoptosis marker cleaved caspase-3 and p-Met expression in tumors. Representative images of tumor cells that stained positive for Ki-67, cleaved caspase-3 and p-Met in tumor tissue sections from control and treated groups are shown. Scale bar, 100 μm.

To evaluate the effect of E7050 on tumor growth in vivo, we established a MES-SA/Dx5-LG cell-derived xenograft mouse model. The mice bearing multidrug-resistant human uterine sarcoma xenografts received an oral dose of vehicle or 50 or 175 mg/kg E7050 once daily for 28 days before using the IVIS Lumina Imaging System. Bioluminescent signals were detected in the MES-SA/Dx5-LG cells to monitor the growth of xenograft tumors, and the results showed that the tumor progression was substantially inhibited in the E7050-treated groups as compared to the vehicle-treated control group (Figure 6A,B). Moreover, the tumors from different groups of mice removed and collected at the end of day 28 are shown in Figure 6C, and the mean volumes of subcutaneous tumors from day 0 to day 28 are provided in Figure 6D. Our results revealed that tumor growth in the E7050-treated groups was remarkedly suppressed in a dose-dependent manner when compared with that in the vehicle-treated group at the end of the experiment. The tumor appearance and size were consistent with the statistical result of tumor volume, indicating that E7050 treatment significantly inhibits tumor growth. The histology of xenograft tumors was also assessed by H&E staining; the data showed that the tumor size was smaller in E7050-treated groups than in the vehicle-treated control group (Figure 7A). To further substantiate in vitro results showing effects of E7050 on proliferation and apoptosis, tumor tissues were assessed by immunohistochemical analysis with Ki-67 and cleaved caspase-3 antibodies. As shown in Figure 7B, E7050 treatment notably decreased the number of proliferating cells (Ki-67-positive cells) in tumors of MES-SA/Dx5 xenografts as compared to the vehicle-treated control. In contrast, the expression of cleaved caspase-3 in the E7050 treatment groups was obviously higher than that in the vehicle control group. In addition, the p-Met-positive cells were markedly reduced in tumors by E7050 treatment, which was similar to the results of our in vitro analysis (Figure 7B). Altogether, these findings suggest that E7050 serves as a c-Met tyrosine kinase inhibitor, which is able to suppress tumor progression in the MES-SA/Dx5 xenograft model.

## 3. Discussion

The anti-cancer drug DOX is widely used for treating several cancers, including uterine sarcoma [2]. However, MDR is the major problem preventing effective cancer chemotherapy. Multidrug-resistant uterine sarcoma MES-SA/Dx5 cells are derived from the cell line MES-SA through prolonged in vitro treatment with DOX [28]. E7050, a c-Met tyrosine kinase inhibitor, has shown anti-tumor effect against lung cancer and gastric cancer [20,29]; nevertheless, its effect on multidrug-resistant uterine sarcoma has not been investigated. In the present study, we found, for the first time, that E7050 is able to reduce the viability of MES-SA/Dx5 cells by the induction of apoptosis and S phase cell cycle arrest.

The abnormal regulation of apoptosis can result in uncontrolled cell division, leading to diseases such as cancer. Thus, inducing apoptosis is regarded as one of major mechanisms for cancer therapy [30]. The main feature of apoptosis is the activation of caspase cascades, which is essential for apoptotic cell death in both intrinsic and extrinsic pathways [31]. Caspase-3, the most critical apoptosis downstream performer of caspase cascade, is known to catalyze the cleavage of PARP and actively induce cellular apoptosis [23]. Our results revealed that E7050 induced apoptosis in MES-SA/Dx5 cells as evidenced by increasing the levels of cleaved caspase-3 and PARP. It has been reported that the tumor suppressor p53 plays a major role in the control of apoptosis [32]. Several p53-inducible genes, which are involved in apoptosis and cell cycle arrest, have been identified, such as *Bax* and *p21* [33,34]. Meanwhile, apoptosis-related proteins, also known as Bcl-2 family members, such as anti-apoptotic protein Bcl-2, and pro-apoptotic protein Bax, also regulate apoptosis [35]. In this study, we found that treating MES-SA/Dx5 cells with E7050 resulted in an elevated level of p53 protein in MES-SA/Dx5 cells. Upon activation of the p53-dependent apoptosis pathway, the pro-apoptotic protein Bax was also upregulated. Moreover, the IAP protein survivin is highly expressed in most cancers, and has been shown to inhibit apoptosis, enhance proliferation and promote angiogenesis [36]. Our study demonstrated that E7050 treatment could result in the downregulation of survivin, which contributes to the anti-cancer effect of E7050 through inhibition of cell proliferation and activation of apoptosis.

Cell cycle deregulation is a common feature of human cancers, and numerous chemotherapeutic drugs exert anti-cancer properties by interfering with the cell cycle [37]. In our research, sub-G1 accumulation of the vehicle-treated cells was 3.2%, which significantly increased up to 9.08% with 25 μM E7050 treatment. The sub-G1 phase had an obvious difference of about 2.8-fold at 25 μM E7050 compared to the vehicle-treated control group (Supplementary Figure S1B). The increasing of the sub-G1 phase population demonstrates that E7050 can cause cellular apoptosis in MES-SA/Dx5 cells. Moreover, MES-SA/Dx5 cells showed a significant increase in S phase after treatment with E7050 for 48 h. As shown in Supplementary Figure S1A, the population of S phase in vehicle-treated cells was 30.1%, and arrested cells increased to 38.59% with 25 μM E7050 treatment. The present study further determined the expression levels of cyclins and other regulatory proteins in MES-SA/Dx5 cells. Treatment with E7050 at 25 μM significantly decreased the expression of cyclin A (S phase-related protein), and increased in the expression of cyclin D1 (G0/G1 phase-related protein) in MES-SA/Dx5 cells. However, the change in expression of cyclin B1 (G2/M phase-related protein) was not clearly related to the change of E7050 concentration (Supplementary Figure S1C–G). Another study by Taniguchi et al. also reported that cyclin A is remarkably decreased in small-cell lung cancer cells by treatment with golvatinib (E7050) [38], which is consistent with the finding in the present study. In addition, the cyclin-dependent kinase (CDK) inhibitor p21 is known to inhibit the kinase activity of cyclin A/CDK1/2 complexes, leading to cell cycle inhibition through and into S phase [39]. P21 can also be regulated by both p53-dependent and p53-independent mechanisms [39]. In our research, the p53 expression was markedly increased by E7050 treatment in MES-SA/Dx5 cells, along with a significant increase in the protein level of p21. Consequently, these results suggest that the downregulation of cyclin A and the upregulation of p21 might be responsible for S phase arrest induced by E7050 in MES-SA/Dx5 cells.

**Figure 8:**
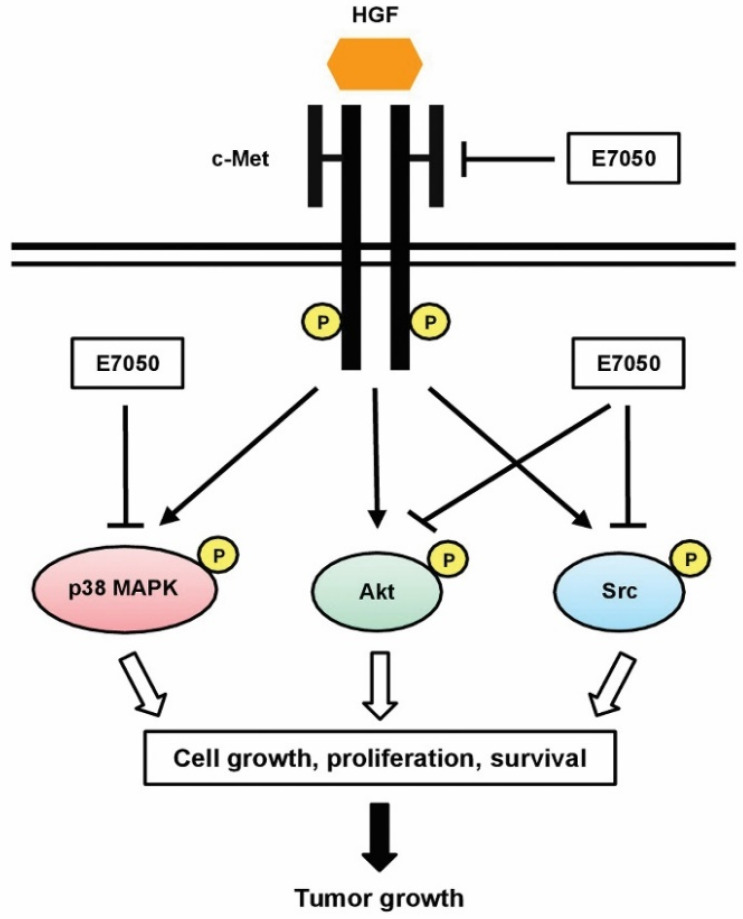
Pathway diagram presenting E7050 mode of action associated with tumor growth inhibition of MES-SA/Dx5 cancer cells. E7050 could inhibit the phosphorylation of c-Met (the receptor of HGF), thereby inhibiting the HGF-activated p38 MAPK, Akt and Src signaling pathways, and further reducing the growth of MES-SA/Dx5 tumor cells.

Since c-Met activation is involved in a number of biological functions, such as cell proliferation, motility and anti-apoptosis, the inhibition of c-Met can induce apoptosis [40,41]. Thus, we detected the expression and phosphorylation levels of c-Met in MES-SA/Dx5 cells. The experimental results revealed that E7050 induced apoptosis in MES-SA/Dx5 cells by inhibiting the activation of c-Met via inhibiting its phosphorylation. The major arm of c-Met signaling, the PI3k/Akt signaling pathway, is involved in the proliferation and survival of cells [14]. In the present study, E7050 treatment effectively suppressed the phosphorylation of Akt, showing that it blocks growth signaling in MES-SA/Dx5 cells. Moreover, Src tyrosine kinase has been suggested as a downstream target molecule in the c-Met cascades, and its inhibition in cancer cells can lead to reduced anchorage-independent growth, proliferation, survival, migration, invasion, metastasis and tumor vascularity [42]. MAPKs are also downstream targets of c-Met signaling pathways, which regulate proliferation, migration and apoptosis of tumor cells [16,43]. Based on our results, when we evaluated other potential targets of E7050, we observed that the phosphorylation of Src and p38 MAPK was eminently attenuated. Furthermore, other investigators demonstrated that the combination of c-Src and c-Met inhibitors results in synergistic cytotoxicity, enhanced apoptosis and decreased tumor size [44]. Previous studies also reported that the p38 MAPK signaling pathway participates in the process of apoptosis in cancer cells, and inactivation of the p38 MAPK signaling leads to the inhibition of proliferation, migration, metastasis and invasion in tumor cells [45,46]. Collectively, these findings of the current study suggest that the downregulation in the phosphorylation levels of c-Met and its downstream signaling kinases Akt, Src and p38 MAPK by E7050 can result in the inhibition of multidrug-resistant cancer cell growth, proliferation and survival (Figure 8).

More importantly, when the experiments were performed in the xenograft mouse model, we observed that E7050 (50 and 175 mg/kg) administration significantly retarded the growth of MES-SA/Dx5 uterine sarcoma xenografts in vivo. Additionally, E7050 administration did not show evident body weight loss and organ toxicity in nude mice bearing MES-SA/Dx5 cell-derived xenografts. As the inhibition of cell proliferation and the induction of apoptosis were observed in E7050-treated MES-SA/Dx5 cells, we further detected the typical reported targets which are involved in proliferation and apoptosis in MES-SA/Dx5 cell-derived tumor tissues of E7050-treated mice. Ki-67, the cell proliferation marker, is widely used as an indicator of prognosis in many cancers [47]. The activated caspase-3, the executor of apoptosis, is a primary target for cancer treatment [48]. The results demonstrated that E7050 obviously suppressed the proliferation and induced apoptosis of multidrug-resistant uterine sarcoma cells in vivo, which agrees with the findings in our in vitro study. Moreover, the expression of p-Met was significantly downregulated in the tumors treated with E7050 in the murine xenograft model. These observations are similar to previous reports, in which blocking c-Met receptor tyrosine kinase signaling transduction by the inhibition of c-Met phosphorylation strikingly repressed tumor growth and metastasis [49].

## 4. Materials and Methods

### 4.1. Reagents and Antibodies

Bovine serum albumin (BSA), dimethyl sulfoxide (DMSO), doxorubicin hydrochloride (DOX), sodium dodecyl sulfate (SDS), Tris buffered saline (TBS), Tween 20 and McCoy’s 5A medium were purchased from Sigma-Aldrich (Saint Louis, MO, USA). Recombinant human hepatocyte growth factor (HGF), fetal bovine serum (FBS), 0.25% trypsin-EDTA, sodium pyruvate, penicillin–streptomycin and phosphate buffered saline (PBS) were purchased from Thermo Fisher Scientific (Waltham, MA, USA). E7050 (golvatinib) was obtained from Selleckchem (Houston, TX, USA). E7050 was dissolved in DMSO at 5 mM concentration as a stock solution and stored at −20 °C in small aliquots. Antibodies against Akt, phospho (p)-Akt (Ser473), ERK1/2, p-ERK1/2 (Thr202/Tyr204), FAK, p-FAK (Tyr397), Met, p-Met (Tyr1349), p38 MAPK, p-p38 MAPK (Thr180/Tyr182), SAPK/JNK, p-SAPK/JNK (Tyr183/Tyr185), Src, p-Src (Tyr416), cleaved caspase-3, PARP and cleaved PARP were purchased from Cell Signaling Technology (Beverly, MA, USA). Antibodies specific to cyclin B1, cyclin D1 and p21 were purchased from Proteintech (Chicago, IL, USA). HGF, Ki-67 and p-Met (Tyr1349) antibodies were purchased from Abcam (Cambridge, MA, USA). Bax, caspase-3, cyclin A, p53, survivin and β-actin antibodies were purchased from Santa Cruz Biotechnology (Dallas, TX, USA). Horseradish peroxidase (HRP)-conjugated goat anti-rabbit and anti-mouse IgG antibodies were obtained from Santa Cruz Biotechnology.

### 4.2. Cell Lines and Cell Culture

Human uterine sarcoma cell line MES-SA was purchased from the American Type Culture Collection (ATCC, Manassas, VA, USA). MES-SA/Dx5 is a multidrug-resistant subline derived from MES-SA. Human umbilical vein endothelial cells (HUVECs) were procured from the Bioresource Collection and Research Center, Food Industry Research and Development Institute (BCRC, Hsinchu, Taiwan). MES-SA/Dx5 cells were maintained in McCoy’s 5A medium supplemented with 10% FBS, 1 mM sodium pyruvate, 100 U/mL penicillin, 100 μg/mL streptomycin and 0.4 μg/mL of DOX. HUVECs were grown in M199 medium containing endothelial growth medium (EGM)-2 SingleQuots Kit (consisting of FBS, VEGF and other growth factors) (Lonza, Basel, Switzerland), 1 mM sodium pyruvate, 10 mM HEPES, 100 U/mL penicillin and 100 μg/mL streptomycin. All the cells were cultured at 37 °C in a humidified incubator with 95% air and 5% CO_2_.

### 4.3. Cell Viability Assay

MES-SA/Dx5 (4 × 10^4^ cells/well) cells were seeded into 96-well plates. After incubation for 24 h, the cells were treated with various concentrations (5, 10, 25 and 50 μM) of E7050 for 24 or 48 h, and 1% DMSO was used as the vehicle control. Cell viability was assessed using the MTT-based In Vitro Toxicology Assay Kit (Sigma-Aldrich) as previously described [50].

### 4.4. Cell Apoptosis Analysis

To measure apoptosis in the MES-SA/Dx5 cells treated with E7050, an Annexin V-FITC Apoptosis Detection Kit was utilized (BioVision, Mountain View, CA, USA). Briefly, cells (1 × 10^6^ cells/well) were inoculated evenly in 6-well plates and incubated overnight for cell adherence. The next day, cells were treated with vehicle or E7050 at 5, 10, 25 and 50 μM for 24 h. After treatment, cells were harvested and washed with ice cold PBS. The collected cells were resuspended with 1X Binding Buffer, followed by addition of Annexin V-FITC and propidium iodide (PI), and the mixture was gently mixed and incubated at room temperature in the dark for 5 min. Afterward, apoptotic cells were analyzed using the FACSCalibur flow cytometer and CellQuest software (BD Biosciences, Franklin Lakes, NJ, USA).

### 4.5. Cell Cycle Analysis

The effect of E7050 on cell cycle distribution in MES-SA/Dx5 cells was determined using the EZCell^TM^ Cell Cycle Analysis Kit (BioVision). Cells were treated with indicated concentrations (5, 10 and 25 μM) of E7050 and cultured for 48 h. Subsequently, cells were harvested by trypsinization, washed with ice cold 1X Cell Cycle Assay Buffer, fixed with ice cold 70% ethanol and put on ice for 30 min. For staining, cells were washed with 1X Cell Cycle Assay Buffer, resuspended with Staining Solution and then incubated at room temperature in the dark for 30 min. Ultimately, cell cycle distribution was measured using the FACSCalibur flow cytometer (BD Biosciences).

### 4.6. Establishment of MES-SA/Dx5 Cells Expressing Luciferase (MES-SA/Dx5-LG Cells)

MES-SA/Dx5 cells were seeded in 12-well culture plates, and the cells were transduced with equal amounts of pLenti-LucEGFP virus particles. The stably transduced cells were designated MES-SA/Dx5-LG as previously described [19].

### 4.7. Tumor Xenograft Model

Eight-week-old male BALB/c nude mice (average weight of 18–22 g) were purchased from the National Laboratory Animal Center (Taipei, Taiwan). Mice were maintained under super pathogen-free conditions and constant temperature and humidity with a 12 h light–dark cycle. Food and water were given ad libitum. All animal experiments conformed to the established guidelines of the Institutional Animal Care and Use Committee of Chang Gung University (Taoyuan, Taiwan). Cultured MES-SA/Dx5-LG cells (1 × 10^6^ cells/mice) were mixed with Matrigel (BD Biosciences) followed by subcutaneous injection into the dorsal region near the thigh of mice. After the tumor size reached a volume of approximately 50 mm^3^, the mice were randomly divided into three groups (*n* = 5 in each group) including a vehicle control (distilled water) group and two E7050 (50 mg/kg and 175 mg/kg, dissolved in 0.1 mL of sterile distilled water) dosage groups. The mice were treated with E7050 or vehicle control by oral gavage once a day. Tumor dimensions were measured every 7 days for up to 4 weeks, and calculated using the following standard formula: tumor volume (mm^3^) = 0.5 × larger diameter (mm) × small diameter (mm^2^). All the mice were sacrificed on day 28 after treatment with E7050, and tumor tissue samples were collected for further analysis.

### 4.8. In Vivo Bioluminescence Imaging

To observe the effect of E7050 on tumor growth of mice bearing uterine sarcoma xenografts stably expressing luciferase, bioluminescence imaging was performed using the IVIS Spectrum In Vivo Imaging System (PerkinElmer, Santa Clara, CA, USA) at day 28 after treatment. D-luciferin (Promega, Madison, WI, USA) was dissolved in PBS (15 g/L) and was administered intraperitoneally at a dose of 10 mg/g of body weight before observation. Mice were anesthetized with 2% isoflurane/oxygen mixture and placed in a chamber for imaging. Every tumor was assigned a region of interest, and the signal was calculated according to the number of photons emitted from the surface (photons/s/cm^2^/steradian).

### 4.9. Histopathology and Immunohistochemistry

Xenograft tumor tissues from control and E7050-treated mice were collected and fixed with 4% paraformaldehyde, then embedded in paraffin and routinely processed. Serial paraffin sections (5 µm thick) were prepared using a microtome, and deparaffinized tissue sections were stained with hematoxylin and eosin (H&E, Sigma-Aldrich), according to standard protocols. Immunohistochemistry analysis of tumor tissue sections was carried out using the EnVision Detection Systems (Dako, Glostrup, Denmark) according to the manufacturer’s instructions. Sections were boiled in 10 mM sodium citrate buffer (pH 6.0) for 10 min to expose antigens, and treated with 3% hydrogen peroxide for 10 min to inactivate endogenous peroxidase activity. After blocking with 5% goat serum, the sections were incubated overnight with primary antibodies against Ki-67 (Abcam), cleaved caspase-3 (Cell Signaling) and p-Met (Tyr1349) (Abcam) at 4 °C, followed by incubation with secondary antibodies provided in a kit for 1 h at 37 °C. Finally, the sections were stained with 3,3′-diaminobenzidine (DAB) substrate, counterstained with hematoxylin and mounted on glass slides. Immunostaining was observed in a blind manner by two experienced pathologists. Images were taken with a HistoFAXS tissue analysis system (Tissue Gnostics, Vienna, Austria).

### 4.10. Enzyme-Linked Immunosorbent Assay (ELISA)

The concentrations of HGF in E7050-treated MES-SA/Dx5 culture medium were measured using a human HGF ELISA Kit PicoKine^TM^ according to the manufacturer’s instructions (Boster Biological Technology, Pleasanton, CA, USA).

### 4.11. Western Blot Analysis

Cells were collected and lysed in RIPA lysis buffer (Millipore, Billerica, MA, USA). Protein extraction and Western blot analysis were performed as previously described [50]. Equivalent amounts of protein from each sample were loaded onto 8% to 12% SDS-polyacrylamide gels. After electrophoretic separation, proteins were electrotransferred onto polyvinylidene fluoride (PVDF) membranes (Millipore). The membranes were blocked with 5% nonfat dry milk or BSA in TBST buffer (Tris buffered saline with 0.1% Tween 20, pH 7.4) for 1 h at room temperature, followed by incubation overnight at 4 °C with the indicated primary antibodies. After washing steps, the membranes were further incubated with the appropriate HRP-conjugated secondary antibodies for 90 min at room temperature. Finally, immunoreactive protein bands were detected using the enhanced chemiluminescence detection system (Millipore), and the relative amount of protein expression was quantified by densitometry.

### 4.12. Statistical Analysis

All the results from at least three different experiments in the text are represented as the mean ± standard error of the mean (SEM). Statistical analysis of the data was evaluated using either one-way analysis of variance (ANOVA) followed by Dunnett’s post hoc test or two-tailed Student’s *t*-test. A *p*-value less than 0.05 was considered statistically significant.

## 5. Conclusions

In this study, we report the effect of E7050 in multidrug-resistant uterine sarcoma and its underlying mechanisms. The results demonstrate that E7050 treatment can significantly inhibit the viability of multidrug-resistant human uterine sarcoma MES-SA/Dx5 cells through inducing apoptotic cell death and cell cycle arrest in S phase. Moreover, E7050 suppresses uterine sarcoma cell growth via inhibiting the phosphorylation of c-Met and its downstream signaling kinases. The anti-tumor activity of E7050 is further strengthened by its inhibitory effect on the growth of MES-SA/Dx5 uterine sarcoma xenografts in vivo. Therefore, E7050 can act as a potent anti-tumor inhibitor with the potential to become an alternative agent for the treatment of multidrug-resistant uterine sarcoma and other types of multidrug-resistant cancers.

## Data Availability

The data presented in this study are available on request from the corresponding author.

## Supplementary Materials

The following supporting information can be downloaded at: https://www.mdpi.com/article/10.3390/ijms232314884/s1.
