# Supplementary material for: Blockade of c-Met-Mediated Signaling Pathways by E7050 Suppresses Growth and Promotes Apoptosis in Multidrug-Resistant Human Uterine Sarcoma Cells
Source: Int J Mol Sci. 2022 Nov 28;23(23):14884. doi: 10.3390/ijms232314884 (PMC9740914; doi:10.3390/ijms232314884)
Supplement: Supplementary file 1 [file ijms-23-14884-s001.zip › ijms-2031991-supplementary.pdf]

Supplementary Materials

Blockade of c-Met-Mediated Signaling Pathways by E7050 Suppresses Growth and Promotes Apoptosis in Multidrug-Resistant Human Uterine Sarcoma Cell

Tsung-Teng Huang, Chuan-Mu Chen, Ying-Wei Lan, Song-Shu Lin, Kong-Bung Choo and Kowit-Yu Chong

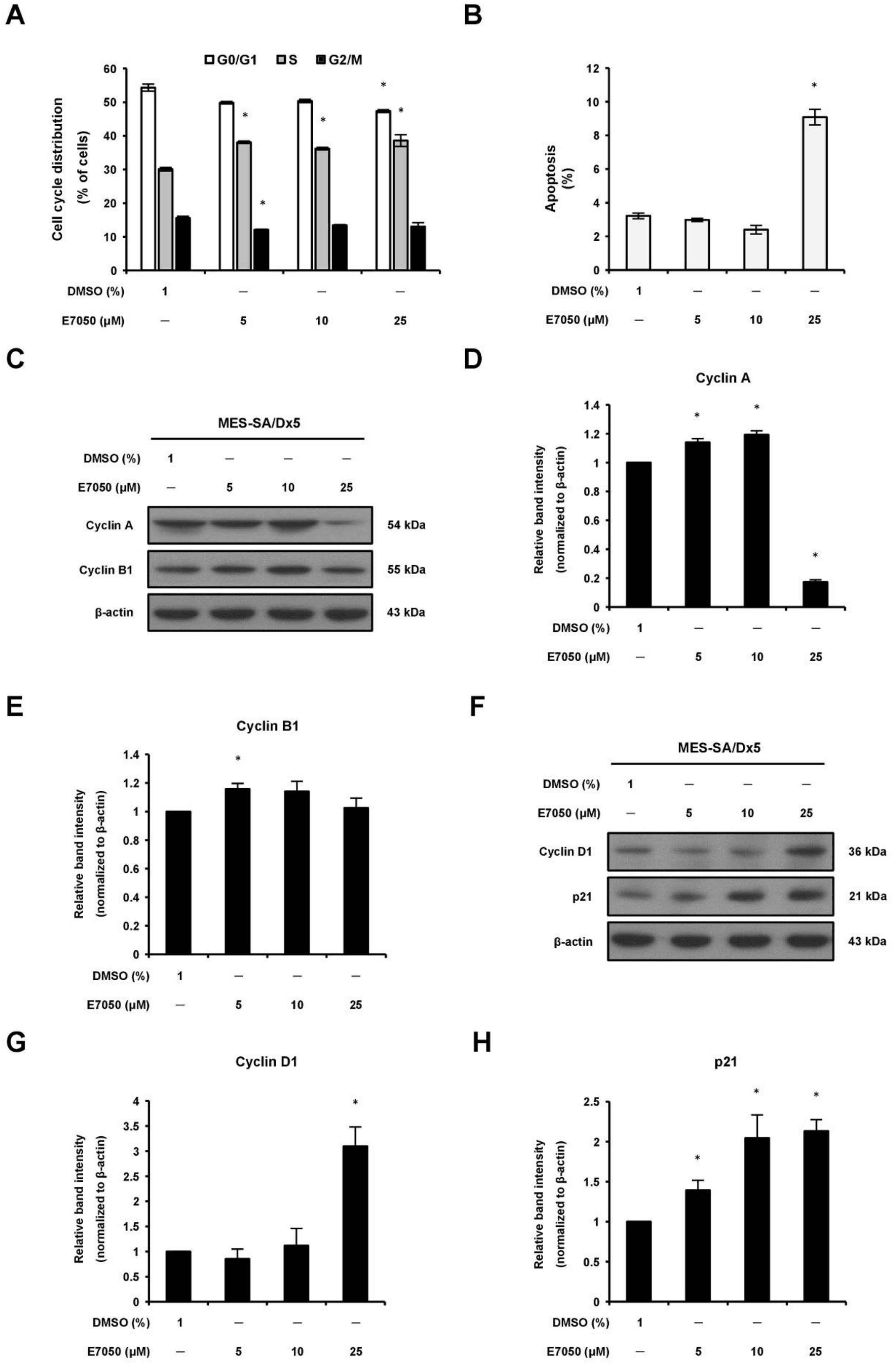

Supplementary Figure S1. Effects of E7050 on cell cycle distribution and the expression of cell cycle regulatory proteins in MES-SA/Dx5 cells. Cells were treated with various concentrations (5–25 μM) of E7050 for 48 h. The cell cycle distribution of

the treated cells was evaluated by flow cytometry. (**A**) The percentages of cells in the G0/G1, S, and G2/M phases were shown. (**B**) The percentage of apoptotic cells (sub-G1 phase) was shown. (**C**) Effect of E7050 on the protein expression levels of cyclin A and cyclin B1 were determined by Western blot analysis, with β-actin as a loading control. The bar graphs represent densitometric analysis of (**D**) cyclin A and (**E**) cyclin B1 protein expression levels after normalized with β-actin. (**F**) The protein expression levels of cyclin D1 and p21 were measured by Western blot analysis. Densitometric analysis of (**G**) cyclin D1 and (**H**) p21 protein expression levels after normalized with β-actin. Data are presented as the mean ± SEM of three independent experiments. *$P < 0.05$ versus vehicle-treated control cells.

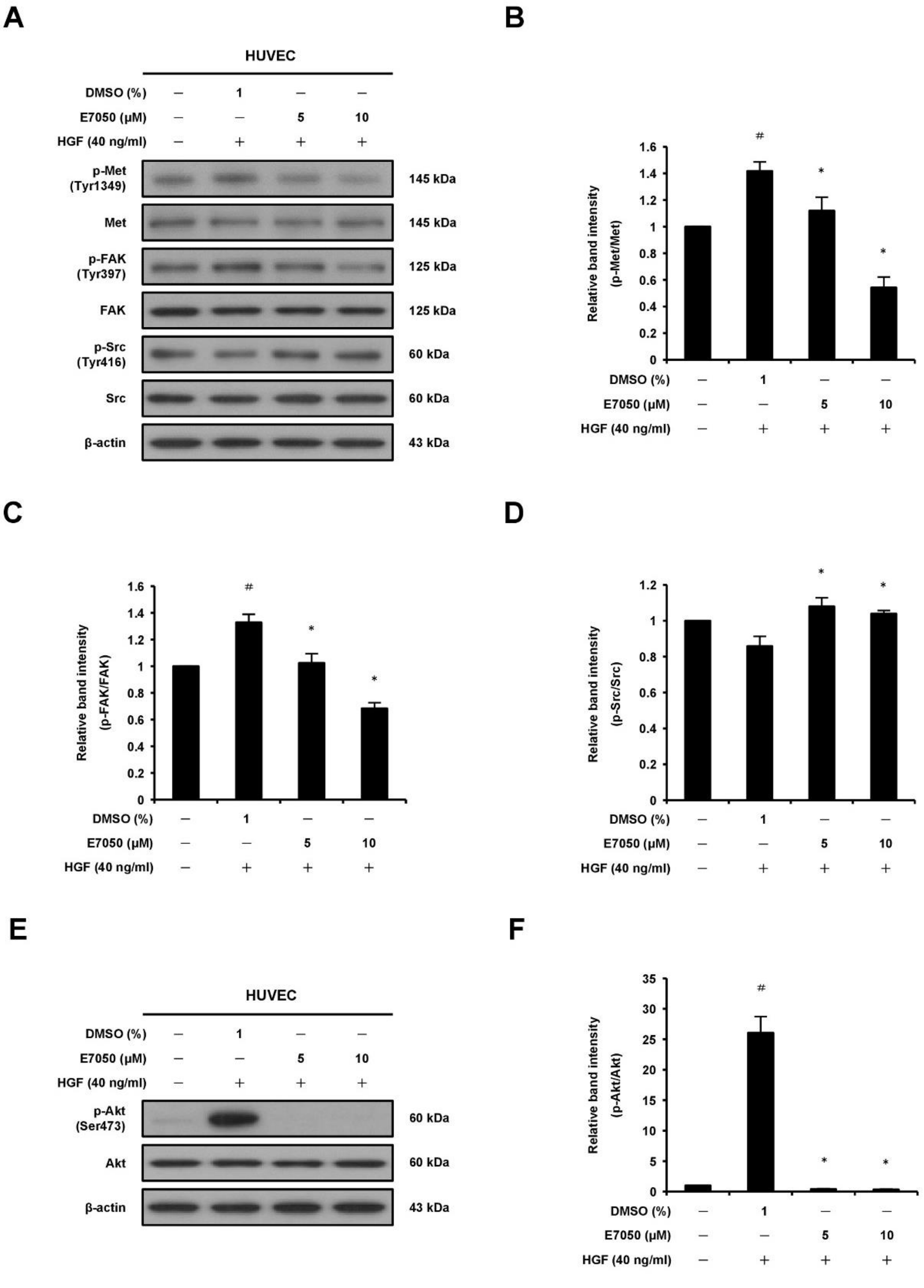

A
HUVEC
DMSO (%) – 1 – –
E7050 (μM) – – 5 10
HGF (40 ng/ml) – + + +
p-Met (Tyr1349) 145 kDa
Met 145 kDa
p-FAK (Tyr397) 125 kDa
FAK 125 kDa
p-Src (Tyr416) 60 kDa
Src 60 kDa
β-actin 43 kDa
B
Relative band intensity (p-Met/Met)
C
Relative band intensity (p-FAK/FAK)
D
Relative band intensity (p-Src/Src)
E
HUVEC
p-Akt (Ser473) 60 kDa
Akt 60 kDa
β-actin 43 kDa
F
Relative band intensity (p-Akt/Akt)

G

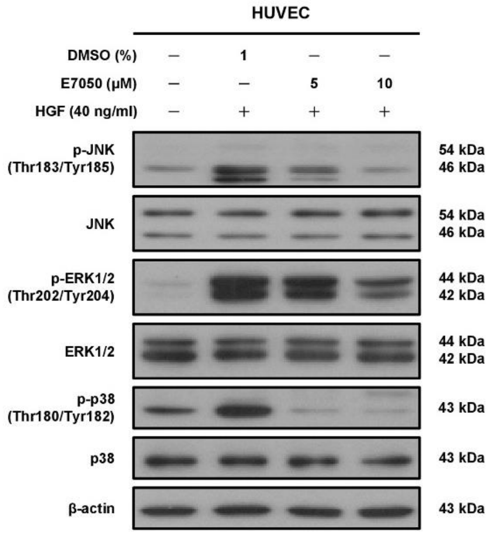

H

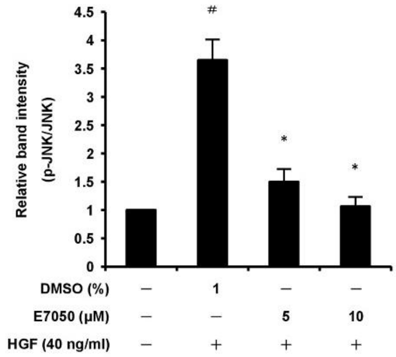

I

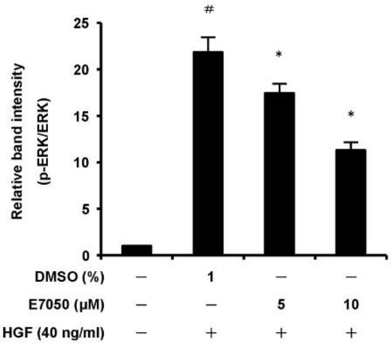

J

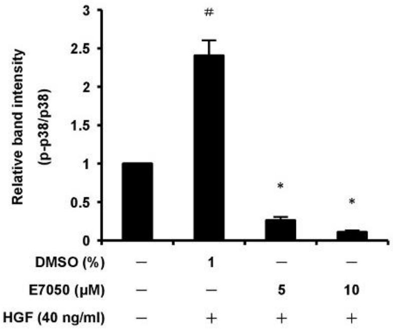

Supplementary Figure S2. Effects of E7050 on the phosphorylation of c-Met and its downstream signaling mediators in HGF-stimulated HUVECs. Cells were starved in serum-free medium for 6 h, pretreated with the indicated concentrations (5 and 10 μM) of E7050 for 1 h, and then stimulated with HGF (40 ng/ml) for 10 min (Met, FAK, and Src) or 30 min (Akt, JNK, ERK, and p38 MAPK) before protein extraction. The expression levels of Met and its downstream intracellular kinases and their phosphorylated forms were measured by Western blot analysis. β-actin was used as an internal loading control. (**A**) E7050 inhibited the phosphorylation of Met and FAK in HGF-induced HUVECs. The images shown are representative Western blotting data. (**B–D**) The ratios between the expression of proteins (Met, FAK, and Src) and their phosphorylated forms (p-Met, p-FAK, and p-Src) were calculated and shown. (**E**) E7050 inhibited the phosphorylation of Akt in HGF-induced HUVECs. (**F**) The ratio between the expression of Akt and p-Akt was calculated and shown. (**G**) E7050 suppressed the phosphorylation of JNK, ERK, and p38 MAPK in HGF-induced HUVECs. The images shown are representative Western blotting data. (**H–J**) The compiled results of the ratios of p-JNK, p-ERK, and p-p38 MAPK normalized to relative protein levels were shown. Data are presented as the mean ± SEM of three independent experiments. $^{\#}P < 0.05$ versus untreated cells. $^{*}P < 0.05$ versus vehicle-treated control cells.
